# Revisiting the efficacy of bismuth subsalicylate for the prevention of traveller’s diarrhoea

**DOI:** 10.1093/jtm/taaf076

**Published:** 2025-07-26

**Authors:** Roger D Gibb, Jose M Brum, Kyle J Sloan, Adam M Pitz, Benjamin T Circello, Rowan A Grayling

**Affiliations:** Healthcare Biostatistics, The Procter and Gamble Company, Mason, OH 45040, United States; Healthcare Clinical, The Procter and Gamble Company, Mason, OH 45040, United States; Healthcare R&D, The Procter and Gamble Company, Mason, OH 45040, United States; Healthcare R&D, The Procter and Gamble Company, Mason, OH 45040, United States; Healthcare R&D, The Procter and Gamble Company, Mason, OH 45040, United States; Healthcare R&D, The Procter and Gamble Company, Mason, OH 45040, United States

**Keywords:** Bismuth subsalicylate, traveller’s diarrhoea

## Abstract

What can be concluded from a clinical trial that spanned 5 years yet failed to achieve target enrollment, generated flat results that contradict the existing body of knowledge and has a primary efficacy endpoint confounded by a treatment-by-COVID interaction? Could it be a false negative finding?

A recent article by Angelo *et al*.^1^ investigated the impact of bismuth subsalicylate (BSS, 2.1 g daily dose) on the prevention of traveller’s diarrhoea (TD). The study began in 2018 and ran for 5 years, which included a COVID pause. Having not achieved its enrollment target in 2023, the study was terminated early (NCT03535272) and failed to find a treatment effect. The purpose of this letter is to share additional perspective on this finding.

Three previously conducted randomized, placebo-controlled, double-blind clinical studies showed BSS to have a statistically significant and clinically meaningful TD prevention effect.^2–4^ The designs of these studies were similar in many respects to that reported by Angelo *et al*., the results of which are summarized in Figure 1. All studies dosed 2.1 g BSS daily except for Dupont (1980)^3^ which dosed 4.2 g BSS daily. Why might Angelo *et al*. have failed to detect a TD preventive effect for BSS when it was so manifestly evident in these three historical studies?

**Figure 1:**
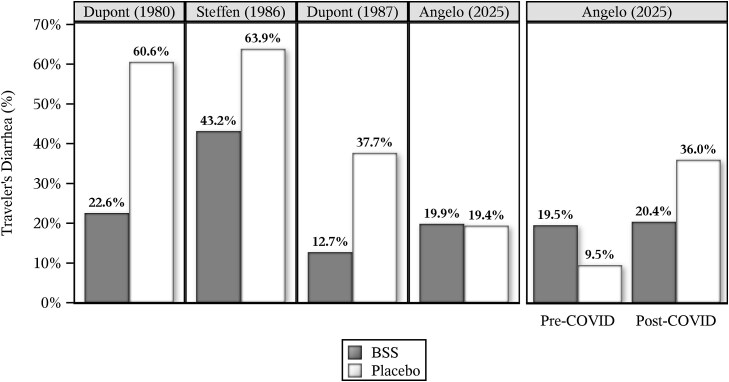
Graphical representation of historical and Angelo *et al*. traveller’s diarrhoea data

One place we might look is subjects’ time of exposure in high risk countries. Angelo *et al*., reported that the median number of foreign travel days was 9.5 and 11 in the placebo and BSS groups, respectively, and this difference was reported by the authors to be statistically significant (*P* = 0.036 in Results section and *P* = 0.053 in Table 2 of Angelo, *et al*.). This 14% exposure bias against BSS may explain, in part, the failure to observe a BSS diarrhoea prevention effect.

The impact of exposure time on TD incidence could also be insightful. A median exposure time of 9.5 days in the placebo group resulted in a TD rate of 19.4%, whereas in the three historical BSS studies exposure was for 21, 12–28 (mean 17.8 days) and 21 days, resulting in placebo diarrhoea rates of 60.6%, 63.9% and 37.7%, respectively. Also, in a recent meta-analysis of 10 cohort studies published between 1997 and 2023, the trip duration ranged from 12 to 22.9 days with a mean of 18.5 days, resulting in TD rates ranging from 27.4% to 49.7%.^5^ The Angelo *et al*. placebo group median trip duration (9.5 days) and TD rate (19.4%) are both well below the aforementioned historical studies, suggesting that the short exposure time may have resulted in the low TD rate. This necessarily curtailed the study’s statistical power to detect a TD preventive effect for BSS.

The data reported in Supplemental Table 2 of Angelo *et al*. show that the TD results pre-COVID and post-COVID are inconsistent (see Figure 1). Pre-COVID the placebo group TD rate was half that of the BSS group (9.5% placebo vs. 19.5% BSS), yet post-COVID this relationship was flipped (36.0% placebo vs. 20.4% BSS). We calculate that this treatment-by-COVID interaction was statistically significant (*P* = 0.013) and was, therefore, unlikely to have happened by chance alone. Had the study ended just before the COVID pandemic, placebo would have strangely been found more effective than BSS. However, had the study begun just after the pandemic started the opposite would have been concluded. Curiously, the TD rate nearly quadrupled from 9.5% pre-COVID to 36.0% post-COVID. The unprecedented vigilance given to infection control associated with the pandemic would be expected to reduce TD, not increase it, as reported by Angelo *et al*. All of these inconsistencies raise questions about the reliability of study results.
